# Quantifying urban heat island intensity in Hong Kong SAR, China

**DOI:** 10.1007/s10661-012-2876-6

**Published:** 2012-09-25

**Authors:** Leong Wai Siu, Melissa A. Hart

**Affiliations:** 1 Department of Geography, The University of Hong Kong, Pokfulam Road, Hong Kong, SAR China; 2 ARC Centre of Excellence for Climate System Science, The University of New South Wales, New South Wales, Australia

**Keywords:** Local climate zone, Urban heat island, Landscape classification, Hong Kong, Meteorological networks

## Abstract

This paper addresses the methodological concerns in quantifying urban heat island (UHI) intensity in Hong Kong SAR, China. Although the urban heat island in Hong Kong has been widely investigated, there is no consensus on the most appropriate fixed point meteorological sites to be used to calculate heat island intensity. This study utilized the Local Climate Zones landscape classification system to classify 17 weather stations from the Hong Kong Observatory’s extensive fixed point meteorological observation network. According to the classification results, the meteorological site located at the Hong Kong Observatory Headquarters is the representative urban weather station in Hong Kong, whereas sites located at Tsak Yue Wu and Ta Kwu Ling are appropriate rural or nonurbanized counterparts. These choices were validated and supported quantitatively through comparison of long-term annual and diurnal UHI intensities with rural stations used in previous studies. Results indicate that the rural stations used in previous studies are not representative, and thus, the past UHI intensities calculated for Hong Kong may have been underestimated.

## Introduction

The year 2009 marked a consequential watershed in human history whereby the number of inhabitants in urban areas exceeded the number of inhabitants in rural areas. In other words, our world transitioned from more rural to more urban (United Nations 2010). Urbanization has brought about modifications of the atmospheric environment on various scales and has extensively changed the surface attributes of urban landscape. The urban heat island (UHI), where air temperatures in cities are greater than those in surrounding rural areas, is a widely documented phenomenon which manifests how humans can alter the atmosphere (Oke 1982). In urban climatology, we pay more attention to how or how much the climate of our environs has changed from its primitive state, rather than simply studying its meteorological conditions, such as temperature and rainfall. For this reason, comparing meteorological elements, between urban and rural areas or between urban and preurban days, has so far been researchers’ most favorable approach when conducting urban climate studies (Oke 1984, 2006a). Despite its popularity, we ought not to overlook its two major limitations.

The first limitation is that it is difficult to determine an urban effect precisely. In his pioneering monograph, Lowry (1977) proposed that an urban effect should be deduced by comparing the contemporary measured values of meteorological elements with their preurban values, which is also known as the urban versus preurban approach. Landsberg (1981), however, commented that this is an idealized approach, and in practice, it is unrealistic to fulfill all the assumptions and data requirements (e.g., scarce preurban meteorological data). On the other hand, Lowry (1977) also outlined other common approaches (e.g., urban–rural differences and upwind–downwind differences) and discussed their corresponding drawbacks. Among these approaches, comparing contemporary urban and rural meteorological elements has long been carried out as the most popular and straightforward method in the literature. However, the assumptions of this approach are even stricter: (1) both urban and rural stations should be on the same contour line of different weather elements; (2) the landscape effect on both stations should be insignificant, and (3) the rural station should have no urban effect. Consequently, we can calculate, at best, an approximate urban effect.

The second limitation lies with the absence of standardized definitions of the terminologies. By way of illustration, the UHI intensity (Δ*T*_urban–rural_), the strength of the most documented urban effect, is conventionally defined as the air temperature difference between the urban and rural sites at a given time (Oke 1982; Arnfield 2003). Stewart and Oke (2006), however, criticized the ambiguous and equivocal meaning of the definitions in UHI studies, in particular, “urban” and “rural,” and questioned the representativeness of weather stations. We cannot validate an urban effect unless we have a set of standards to follow.

We also encounter other challenges. For example, researchers generally have limited choices of sites in a region due to the limited number of meteorological stations, uncertainty in the data, and limited time periods for which data are available (Stewart and Oke 2006). We should be concerned with the repercussions of the above limitations, which may give rise to the underestimation or overestimation of the urban effect, the difficulty of rational comparison between the studies, and hence, the weakened reliability of the literature.

Seeing that the urgency of better communication and transferability among different fields and disciplines is stressed, researchers have developed several standardized urban and rural landscape classification systems (Oke 1984, 2006b). A landscape classification system should aim to categorize any terrain rationally and sensibly by implementing specific criteria related to surface properties. Auer (1978) developed the first landscape classification system adapted into urban climate research. However, instead of surface features, the system is primarily concerned with land features (Stewart 2007). Wanner and Filliger (1989) formulated a simple system for urban sites based on their neighboring orographic features. Ameliorating Ellefsen’s Urban Terrain Zones (UTZ; 1990/1991), Oke (2006a, b) developed the Urban Climate Zones (UCZ) classification which is capable of categorizing landscapes into different zones with increasing levels of influence to the urban climate. As the UCZ is less effective for classifying rural landscapes, Stewart and Oke (2009) expanded it into a more comprehensive system called Local Climate Zones (LCZ). It aims at categorizing the landscape “universe” into 19 local climate zones from four landscape series (city, agricultural, natural, and mixed) according to surface cover, surface structure, and cultural activity. As this system represents a better set of criteria in classifying weather stations and is compatible with the previous systems (e.g., UCZ and UTZ), it has been adopted in several studies (e.g., Saaroni and Ziv 2010) and has been supported by numerical modeling (e.g., Krayenhoff et al. 2009).

Located at the entrance of China’s Pearl River Delta, Hong Kong SAR (22°15′ N, 114°10′ E) has long been recognized as one of the world’s most densely populated and developed cities. Occupied with a population of more than seven million in an area of 1,104.4 km^2^, the region has a humid subtropical climate (Köppen classification, Cwa) (Lands Department 2010). The territory, contrasted with its coastal location, is lacking in flat land. The hilly topography has led to a remarkably high development density in the few areas of relatively shallow slope (Ho 2003). Hong Kong’s urban climate has received wide attention from local researchers (Lam 2010). The majority of these previous studies evaluated the urban effect on Hong Kong’s climate by comparing the meteorological data between various weather stations which are under the administration of the Hong Kong Observatory (HKO). Table 1 shows that previous studies have used a wide range of sites to represent the rural, suburban, or nonurbanized climate of Hong Kong. However, we have following concerns on the choices made.

**Table 1:** Classification of the weather stations in Hong Kong in selected studies

Reference	Urban station	Suburban station	Rural station
Kalma et al. (1978)	HKO, KP		CCH, WGL
Chan and Ng (1991)	HKO		LFS, SHA, TKL, TUN
Stanhill and Kalma (1995)	HKO		WGL
Leung and Ng (1997)	HKO		HKS, LFS, SHA, SKG, TKL, TUN, WGL
Giridharan et al. (2004, 2005, 2007, 2008)			HKS
Leung et al. (2004)	HKO		LFS, TKL
Fung et al. (2009)	HKO	TKL	
Memon et al. (2009)	HKO	CCH, LFS, SHA, SLW	TKL
Yim and Ollier (2009)	HKO		WGL
Lam (2010)	HKO		LFS, TKL

*CCH* Cheung Chau, *HKO* Hong Kong Observatory Headquarters, *HKS* Wong Chuk Hang, *KP* King’s Park, *LFS* Lau Fau Shan, *SHA* Sha Tin, *TKL* Ta Kwu Ling, *TUN* Tun Mun Government Offices, *WGL* Waglan Island

Firstly, there is almost no dispute about the use of the HKO Headquarters as an urban station; however, due to the rapid development in both Hong Kong Island and the Kowloon Peninsula, other stations in these urban areas should receive our attention again. Secondly, there have been numerous rural stations implemented in previous studies, and many studies did not include metadata of the stations used. Rationale behind the choices of meteorological sites used in past urban climate studies of Hong Kong concentrated on the environment and history of a site only. For example, Stanhill and Kalma (1995) considered Waglan Island (WGL) a good choice of rural reference site because its environment is not altered by urban atmospheric pollution and anthropogenic heat generation. Finally, no study has attempted to classify the sites in Hong Kong by any landscape classification systems. Therefore, we suspect the reliability of these stations and the UHI intensities reported in the previous studies.

This study focuses on the methodological concerns in quantifying urban heat island intensity using Hong Kong as a case study. The objectives of this study are as follows: (1) to classify the weather stations from the Hong Kong Observatory’s extensive network of meteorological sites using Stewart and Oke’s LCZ landscape classification system, (2) to identify the representative urban and rural weather stations in Hong Kong from the results of LCZ system, and (3) to validate our choices by investigating the corresponding UHI intensity.

## Materials and methods

### Site selection

The Hong Kong Observatory is the official authority in Hong Kong providing a broad range of meteorological services. It has been operating for more than 120 years except for temporary service interruption during the Second World War and provides the largest weather station network in the region. By the end of 2008, the Observatory had constructed 73 weather stations in its network (Hong Kong Observatory 2009).

The following criteria were utilized to select appropriate weather stations to be included in our classification and subsequent analyses. Firstly, the station must have a sufficiently long history. The station history is associated with the temporal homogeneity of data, which ensures that the data variations are solely due to the weather and climate and provides a trustworthy basis for climate research, applications, and user services (Aguilar et al. 2003; World Meteorological Organization 2010). As most stations were built after the launch of the Automatic Weather Station Network project in 1984, we specified a 10-year minimum requirement for the operational history of a station.

Secondly, the station should possess sufficient instruments for measuring common surface meteorological elements. Not all stations within the network measure all common elements. A few stations are required to report the spatial changes of surface pressure in a region, whereas more stations are necessary for wind and precipitation measurements. It is noteworthy that there are 16 stations measuring rainfall only and 17 measuring wind only in the Observatory network.

Thirdly, the variations of the altitude among the stations should not be too large. The highest elevation of all stations is 955 m, which could cause a roughly 6.5 °C difference from sea level under the standard environmental lapse rate, making it difficult to compare the weather conditions between stations at high elevations and stations closer to sea level.

**Fig. 1:**
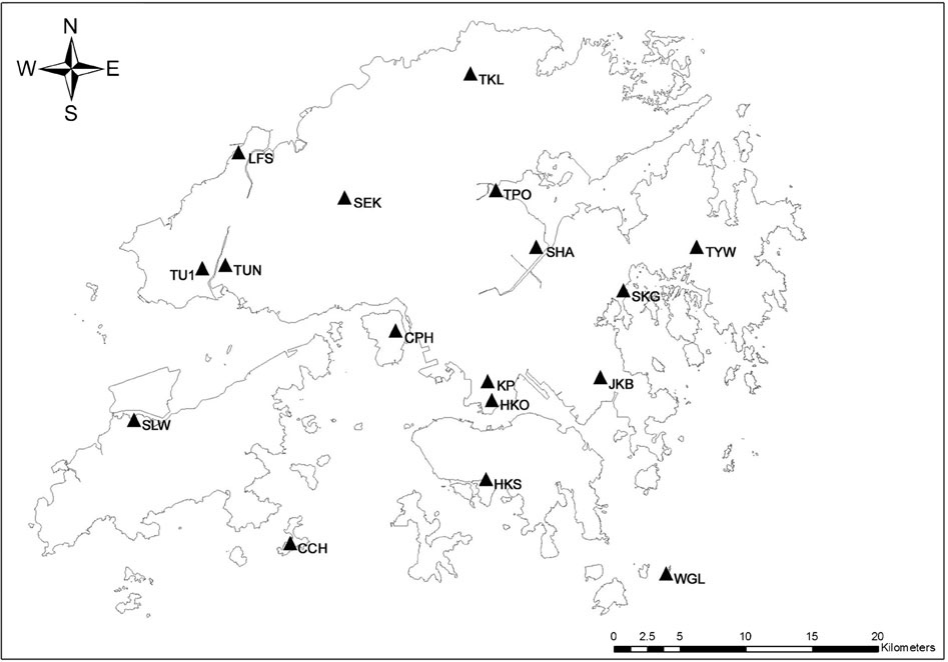
The distribution of the selected weather stations in this study. *HKO* Hong Kong Observatory Headquarters, *TUN* Tuen Mun Government Offices, *CPH* Ching Pak House, *SHA* Sha Tin, *LFS* Lau Fau Shan, *HKS* Wong Chuk Hang, *KP* King’s Park, *TU1* Tuen Mun Children and Juvenile Home, *SKG* Sai Kung, *SEK* Shek Kong, *JKB* Tseung Kwan O, *TPO* Tai Po, *TKL* Ta Kwu Ling, *CCH* Cheung Chau, *TYW* Tsak Yue Wu, *SLW* Sha Lo, *WGL* Waglan Island

Finally, we include that the stations were adopted in the past urban climate studies. As a result, we selected 17 stations according to the above criteria. Temperatures at all 17 stations are aspirated and measured within a Stevenson screen. Table 2 summarizes the geographical characteristics and variables measured at the selected stations; Fig. 1 illustrates their distribution.

**Table 2:** Geographical characteristics of selected weather stations

Station name	Station code	WMO code	Location		Elevation above mean sea level (m)			Date of first operation	Data availability							
			Latitude, N	Longitude, E	Barometer	Anemometer	Ground		W	RF	T	WB	D	RH	P	C
Hong Kong Observatory Headquarters	HKO	45005	22°18′07″	114°10′27″	40	74	32	2 Mar. 1883	†	†	†	†	†	†	†	†
Cheung Chau	CCH	45044	22°12′04″	114°01′36″	80	98	72	1 Jan. 1953	†	†	†	†	†	†	†	
Ching Pak House	CPH	NA	22°20′53″	114°06′33″	–	136	122	1 Apr. 1987	†	†	†	†	†	†		
King’s Park	KP	45004	22°18′43″	114°10′22″	66	90	65	1 Jun. 1951	†	†	†	†	†	†	†	
Lau Fau Shan	LFS	45035	22°28′08″	113°59′01″	36	50	31	16 Sep. 1985	†	†	†	†	†	†	†	
Sai Kung	SKG	45040	22°22′32″	114°16′28″	–	31	4	3 Mar. 1993	†		†	†	†	†		
Sha Lo Wan	SLW	45042	22°17′28″	113°54′25″	52	71	61	25 Feb. 1993	†	†	†	†	†	†	†	
Sha Tin	SHA	45039	22°24′09″	114°12′36″	13	16	6	1 Oct. 1984	†	†	†	†	†	†	†	
Shek Kong	SEK	NA	22°26′11″	114°05′05″	25	26	16	4 Nov. 1996	†	†	†		†	†	†	
Ta Kwu Ling	TKL	45032	22°31′43″	114°09′24″	14	28	15	14 Oct. 1985	†	†	†	†	†	†	†	
Tai Po	TPO	45037	22°26′45″	114°10′44″	16	–	15	3 Feb. 1999			†	†	†	†	†	
Tsak Yue Wu	TYW	NA	22°24′11″	114°19′24″	–	23	5	1 Oct. 1995	†	†	†	†	†	†		
Tseung Kwan O	JKB	45041	22°18′56″	114°15′20″	–	52	38	1 Dec. 1991	†	†	†	†	†	†		
Tuen Mun Children and Juvenile Home	TU1	NA	22°23′09″	113°57′51″	–	–	28	1 Jan. 2007		†	†	†	†	†		
Tuen Mun Government Offices	TUN	45038	22°23′26″	113°58′36″	–	69	63	23 Oct. 1987	†							
Waglan Island	WGL	45045	22°10′56″	114°18′12″	60	83	56	1 Dec. 1952	†	†	†	†	†	†	†	
Wong Chuk Hang	HKS	45043	22°14′54″	114°10′15″	–	30	5	1 Aug. 1989	†		†	†	†	†		

The instruments measuring air temperature and rainfall at TUN were relocated to TU1 on 1 Jan. 2007. TUN continues the wind measurement only (Hong Kong Observatory 2009)

*W* wind, *RF* rainfall, *T* air temperature, *WB* wet bulb temperature, *D* dew point temperature, *RH* relative humidity, *P* mean sea level pressure, *C* cloud amount, *NA* not available

† indicates which variables are measured at each site

### Field observations

Most of the metadata of weather stations were obtained from field observations. After the site selection, we consulted with the staff of the Hong Kong Observatory for planning and evaluating the field trips. Nine field trips were conducted between 2009 and 2011, and 15 stations were visited.

During each trip, the local environment and site characteristics, such as land use, instrument exposure, and site condition, were documented. Photographs were also taken as a record. Aerial photographs covering each meteorological site were used to determine the proportion of water, green space, and built environment surrounding each station. Sky view factor (SVF) was calculated using the RayMan model (Matzararkis et al. 2007) from fish-eye lens photographs taken at each site.

It was not possible to visit two stations: Shek Kong (SEK) and Waglan Island (WGL). The SEK station is situated inside the Shek Kong Military Barracks, where it is difficult to obtain a permit to visit, and photography is not allowed. The WGL station is located at the Waglan Lighthouse on a very small island 5 km off the coast of Hong Kong Island. As the lighthouse became one of the declared monuments in Hong Kong in 2000, it requires a written application to visit, in addition to an often quite risky boat trip.

### Geographic information system

Geographic information system was used to analyze the changes in land use in the Kowloon Peninsula over the last 40 years and the spatial variation of present land use surrounding each weather station. The following land use maps were obtained from the Lands Department and Planning Department.

- Land Utilization in Hong Kong in 1966, 1978, and 1987
- Various Outline Zoning Plans in Hong Kong in 2007

Since land use categories in the land use maps have changed over time, for the sake of comparison, we simplified and unified the categories into the following groups: commercial and residential (many residential buildings in Hong Kong are classified as mixed use because of commercial premises on the ground floor); government, institution, and community facilities; industrial; road or railway; green space; and miscellaneous.

### Determination of the representative urban and rural stations

After the data collection, a “circle of influence,” which was of 250-m radius (Sakakibara and Matsui 2005), was specified to each selected weather station. Every sampled region was then matched with the Local Climate Zones system from the zone description and properties (Stewart and Oke 2009).

Although there has been no universally accepted method to choose representative urban and rural stations, the World Meteorological Organization (WMO) has published a number of guidelines (e.g., Plummer et al. 2003; World Meteorological Organization 2008, 2010) which recommend useful principles on how to establish suitable sites in urban and rural areas. These guidelines shed light on how to select the appropriate sites from the current station network. Ideally, urban and rural stations should monitor the areas which respectively give the largest and least impacts to a city. The results of the local climate zone designation could allow us to pinpoint the representative stations.

### Calculation of the UHI intensity in Hong Kong

Nine stations, which include Hong Kong Observatory Headquarters (HKO), King’s Park (KP), Ta Kwu Ling (TKL), Lau Fau Shan (LFS), Wong Chuk Hang (HKS), Tsak Yue Wu (TYW), Sha Lo Wan (SLW), Cheung Chau (CCH), and Waglan Island (WGL), were chosen to be studied. The rationale behind their inclusion is their popular use in the literature or their representativeness as an urban or rural site. The meteorological data of these weather stations were obtained from the Hong Kong Observatory. The data include both 5-year (2004–2008) hourly and 20-year (1989–2008) daily surface meteorological data. The UHI intensity in Hong Kong was then calculated using the designated urban and rural stations.

## Results and discussion

### Local climate zones

#### Local climates zones of the weather stations in Hong Kong

As shown in Table 3, the LCZ scheme satisfactorily classified all but one of the selected weather stations in Hong Kong into their respective zones. WGL is the only station that could not be classified due to its atypical marine environment. There are total seven different zones in Hong Kong, and all of which are under the city and natural series. The Dispersed Midrise Zone (BCZ9) is the largest group which contains six stations.

**Table 3:** Results of the Local Climate Zone designation of selected weather stations in Hong Kong

Station name	Station code	LCZ series	LCZ (code)	Surface properties in circle of influence			Distance to HKO (km)	Nearest large water body (distance)
				Proportion built (%)	Proportion green (%)	Proportion water (%)		
Hong Kong Observatory Headquarters	HKO	City	Compact High-Rise (BCZ1)	80.0	20.0	0.0	NA	Victoria Harbour (1 km)
Tuen Mun Government Offices	TUN	City	Open-Set Blocks (BCZ5)	74.1	25.9	0.0	23	Castle Peak Bay (1 km)
Ching Pak House	CPH	City	Open-Set Blocks (BCZ5)	72.2	27.5	0.3	9	Rambler Channel (0.3 km)
Sha Tin	SHA	City	Extensive Low-Rise (BCZ6)	57.9	25.6	16.5	12	Sha Tin Hoi (1 km)
Lau Fau Shan	LFS	City	Lightweight Low-Rise (BCZ8)	42.3	30.4	27.3	27	Deep Bay (0.05 km)
Wong Chuk Hang	HKS	City	Dispersed Midrise (BCZ9)	56.6	43.4	0.0	6	Deep Water Bay (1 km)
King’s Park	KP	City	Dispersed Midrise (BCZ9)	47.4	52.6	0.0	1	Victoria Harbour (1.5 km)
Tuen Mun Children and Juvenile Home	TU1	City	Dispersed Midrise (BCZ9)	39.5	60.5	0.0	23	Castle Peak Bay (1 km)
Sai Kung	SKG	City	Dispersed Midrise (BCZ9)	33.3	15.0	51.7	13	Sai Kung Hoi (0.02 km)
Shek Kong	SEK	City	Dispersed Midrise (BCZ9)	32.6	67.4	0.0	18	Deep Bay (8 km)
Tseung Kwan O	JKB	City	Dispersed Midrise (BCZ9)	29.6	70.4	0.0	8.5	Junk Bay (1.5 km)
Tai Po	TPO	City	Dispersed Low-Rise (BCZ10)	20.2	31.9	47.9	16	Tolo Harbour (0.04 km)
Ta Kwu Ling	TKL	City	Dispersed Low-Rise (BCZ10)	18.1	81.9	0.0	25	Sha Tau Kok Hoi (5.5 km)
Cheung Chau	CCH	City	Dispersed Low-Rise (BCZ10)	13.1	79.2	7.7	19	West Lamma Channel (0.2 km)
Tsak Yue Wu	TYW	Natural	Forest (NCZ1)	2.7	96.7	0.6	19	High Island Reservoir (1 km)
Sha Lo Wan	SLW	Natural	Forest (NCZ1)	0.5	75.2	24.3	26	Hau Hok Wan (0.2 km)
Waglan Island	WGL	NA	NA	3.0	18.4	78.6	19	Pacific Ocean (0.07 km)

#### Weaknesses of classifying weather stations in Hong Kong, using the LCZ system

Stewart and Oke (2009) stated that landscape classification system is only a “simplification of the fact.” One should treat the process as a quick and experienced judgment instead of an automated matching owing to the complexity of the actual environment. The complex topography and urban terrain in Hong Kong gives rise to a demanding and arduous task when classifying the weather stations in this region. We noticed four general issues during the classification process.

The first issue occurs when a station is located in the middle between two local climate zones. For example, although Ching Pak House (CPH) station is situated in a public housing estate, the northeastern part of its circle of influence is a large industrial center (Tsing Yi), and the nearest container terminal is within a distance of 600 m. These buildings and construction, according to the LCZ, should belong to Extensive Low-Rise Zone (BCZ6). We finally justified the site as Open-Set Blocks Zone (BCZ5) since the altitude of all industrial buildings is considerably lower than CPH.

The second issue associates with the heterogeneous land use inside the circle of influence of a site. For example, located on a hill in urban Kowloon, King’s Park station is surrounded by one of the largest pieces of urban green space in Hong Kong. The station enjoys a higher sky view factor (SVF = 0.83) because of its higher altitude (65 m), whereas HKO, located only 1 km south from KP, experiences a less visible sky (SVF = 0.44). We finally justified the site as Dispersed Midrise Zone (BCZ9) owing to its large sky view factor and the larger green space proportion (52.6 %). Another example, Lau Fau Shan station is located on a small hill, where we can see a combination of car parks, open storage yards, seafood restaurants, and fishing villages inside its circle of influence. We finally classified the site into Lightweight Low-Rise Zone (BCZ8) due to the mixture of detached and semidetached buildings and narrow streets common in Hong Kong’s fishing villages. However, it should be noted that the weather station is located 200 m inland from the coast and would thereby receive marine influences.

The third issue couples with the inappropriate setting of weather stations. In Hong Kong, most improper settings are related to the location of the station. For instance, CPH station is located on the rooftop of a public 24-story residential building. In addition, all the instruments of Tuen Mun Government Offices (TUN) station were placed on the rooftop of a government building before 1 January 2007. World Meteorological Organization (2008) stated that it is improper for setting up Stevenson screens on rooftops for the following reasons: first, rooftops experience a different microclimate than the surface. The airflow on rooftop contrasts with the surrounding, perturbing wind speed, direction, and gusts. Second, the rooftop material is usually thermally extreme. As a result, there could be an abrupt change of air temperature gradient. Third, the rooftop is usually impermeable so that water can be discharged easily and, hence, results in abnormal dryness on rooftop. CPH was replaced by a new “Tsing Yi” station in April 2011, which is located at ground level. Of an additional concern is the distance between a station and its surrounding obstacles, in particular, trees, buildings, and walls. Three stations, Wong Chuk Hang (HKS), Tai Po (TPO), and Cheung Chau (CCH), are under the shade of neighboring tall trees. In addition, the flat land in Hong Kong is scarce and is often located on the seashore so that, in this study, seven stations are situated within a distance of less than 300 m to their nearest large water body (Table 3).

The final issue concerns classifying sites located on islands. In this study, WGL and CCH are on Waglan Island and Cheung Chau Island, respectively. Table 4 shows that the two islands are quite different with respect to their area and population, thereby having dissimilar results under the LCZ. While the metadata demonstrate that CCH conforms to Dispersed Low-Rise Zone (BCZ10), we could not match WGL with any climate zones because its surrounding ocean constitutes the major proportion of the circle of influence.

**Table 4:** Comparison between Waglan Island and Cheung Chau station

	WGL station	CCH station
Area of the island (km^2^)	0.1	2.4
Population of the island	0	23,200
Proportion built in the circle of influence (%)	3.0	13.1
Proportion water in the circle of influence (%)	78.6	7.7

### Representative urban and rural weather stations in Hong Kong

#### Representative urban weather station

According to the zone designation, HKO is the best choice as a representative urban site in Hong Kong because it is the only site fitting into Compact High-Rise Zone (BCZ1). The proportion of built area (80.0 %) inside its circle of influence is the highest among all the stations. Since the LCZ system is principally concerned with the existing spatial variation of land use, we also explored the temporal urban development for the last 40 years using geographic information system.

As shown in Table 5, the land area of the Kowloon Peninsula has been increasing since 1966, due to the reclamation of land from Victoria Harbour; however, the proportion of developed area (all land uses except green space) still exceeds 90 % in all four maps. It suggests that Kowloon Peninsula has continued to serve as a core urban area for more than 40 years, and therefore, HKO has been functioning as an urban weather station for more than 40 years. However, one must notice that the reclaimed land complicates the evaluation of the effect of surrounding water bodies. The KP station is also located in urban Kowloon, but its microclimatic and local climatic environment is completely different from the HKO station.

**Table 5:** Land utilization of the Kowloon Peninsula over the last 40 years

Type of land use	1966		1976		1988		2007	
	Area (km^2^)	(%)	Area (km^2^)	(%)	Area (km^2^)	(%)	Area (km^2^)	(%)
COM/RES	3.08	31.7	3.16	30.7	3.70	34.3	3.97	26.9
GOV	1.82	18.7	1.23	12.0	1.45	13.4	1.98	13.4
IND	0.92	9.5	0.77	7.5	0.36	3.4	0.06	0.4
R/RW	1.46	15.1	3.51	34.2	2.55	23.6	4.81	32.7
GS	0.31	3.2	0.63	6.2	0.73	6.8	0.16	1.1
Misc	2.11	21.7	0.97	9.4	2.00	18.5	3.75	25.4
Total	9.70	100.0	10.28	100.0	10.79	100.0	14.72	100.0

*COM/RES* commercial and residential; *GOV* government, institution, and community facilities; *IND* industrial; *R/RW* road or railway; *GS* green space; *Misc* miscellaneous

**Fig. 2:**
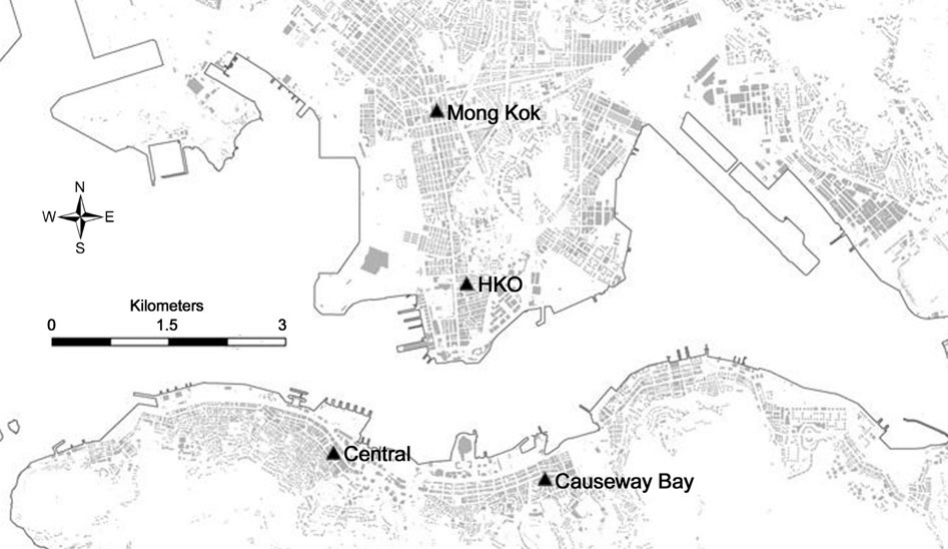
The urban areas of Hong Kong Island and Kowloon Peninsula

Choosing HKO as a representative urban station concurs with all previous studies (e.g., Leung et al. 2004; Fung et al. 2009; Lam 2010), which favor it for similar reasons, such as its long history and constant site exposure. However, one should also be aware of its disadvantages. Firstly, the station is situated close to a large urban woodland located in the HKO headquarters, making its environment markedly divergent from the surrounding high buildings. Secondly, although the site is near a major tourism and trading hub in Hong Kong, many other parts of the urban area also fit into the Compact High-Rise Zone (BCZ1) as well. For instance, being a central business district, the region of Central (Fig. 2), possesses most of the tallest buildings in the city. Causeway Bay (Fig. 2) is another predominantly commercial core on Hong Kong Island. In urban Kowloon, Mong Kok (Fig. 2) is one of the most densely populated areas in the world with an amalgam of commercial and residential buildings (Ingham 2007). From a high-quality and cloud-free satellite image, Fung et al. (2009) pointed out that Mong Kok and Causeway Bay, rather than HKO, are the hottest urban areas in Hong Kong; unfortunately, neither of these locations contain a meteorological site. In short, by relying on the HKO station, both drawbacks would probably underestimate the urban effect in Hong Kong.

#### Representative rural weather station

Compared with the urban sites, there are more nominees for the representative rural stations in Hong Kong: Cheung Chau, Wong Chuk Hang, Lau Fau Shan, Ta Kwu Ling, Waglan Island, Sha Lo Wan, and Tsak Yue Wu. These seven sites can be further divided into two main groups. The first group consists of the first five sites which are popular choices for a representative rural station in the literature (Table 1). However, none of these sites fit into any zones in the natural series of the LCZ; they all fall within the city series.

The CCH station was classified into Dispersed Low-Rise Zone (BCZ10). As Cheung Chau is the most densely populated island in Hong Kong (Table 4) and most residents live on the central part of the island, the station was built on the southern hilly area of the island, making itself nearly the highest point on the island. Its natural exposure and short distance to the sea (200 m) also make it appropriate for being an aeronautical and signal station in the past. Its mean wind speed is among one of the highest weather stations in Hong Kong (Hong Kong Observatory 2009).

The HKS station was classified into Dispersed Mid-Rise Zone (BCZ9), owing to the heterogeneous land use inside its circle of influence. The station is situated within the sports grounds of Hong Kong Police Training School; some industrial and recreational buildings are also within the circle of influence, making the proportion of built area 56.6 %. As a circle of influence shows that how the instruments “see” the surrounding environments, the diverse land use affects how the surface properties affect the instruments (Oke 2006a). Therefore, a site can only be considered representative if the surface properties inside the circle of influence are rather uniform (Stewart and Oke 2010). We also suspect that microclimatic factors influence the site since it is entirely under the shade of surrounding trees.

The LFS station was classified into Lightweight Low-Rise Zone (BCZ6). Leung et al. (2007) discussed the changing environment of this site by stating that the surrounding grassland has been turned into cargo storage yards. From the satellite image, we computed the proportion of green space in its circle of influence, which is only around 30 %. Moreover, it suffers from the influence of sea breeze due to its coastal position. Sakakibara and Owa (2005) indicated that the coastal site could largely underrate UHI intensity in the summer days.

The TKL station was classified into the Dispersed Low-Rise Zone (BCZ10) due, in large part, to its nearby villages. Although previous studies favored this rural site for its rather long history among all weather stations in Hong Kong and stable local environment, the site is located only 5 km from Shenzhen, China. Since the late 1970s, Shenzhen has developed into the world’s largest manufacturing base and is now home to over nine million people which have strikingly surpassed the population of Hong Kong (United Nations 2010). Whereas the distance between TKL and HKO is around 25 km, the distance from TKL to the nearest border of Shenzhen is less than 2 km. Moreover, the distance from TKL to Luohu District, the financial and trading center of Shenzhen, is approximately 5 km. Leung et al. (2007) also recognized that the urban effect from Shenzhen could not be neglected.

The WGL station could not be classified into any climate zones because it is a marine station. As an island, it would inevitably be under the influence of mesoscale sea breeze. In addition, it is difficult to isolate from the local scale urban effect (Goldreich 1984). Moreover, under an intercity study, it would be meaningless to compare the urban effect between a coastal city using a marine rural site and an inland city using an inland rural site.

The natural series of LCZ are those sites with the least urban impact, so the second group includes SLW and TYW which belong to the natural series. Both stations have not been utilized and discussed in previous studies.

The SLW station is designated as Forest Zone (NCZ1). However, we bear in mind that this site is under the influence of sea breeze due to its seashore position. Furthermore, since the 1980s, the Rose Garden Project, which includes the new Airport in Chek Lap Kok, has brought radical changes to its nearby environment (Plant et al. 1998). For example, the reclaimed land of the airport site obstructs the view of SLW, and nowadays, there is only a narrow channel, less than 500 m across, between Sha Lo Wan and the airport.

The TYW station is designated as Forest Zone (NCZ1). Its sky view factor is reasonable (SVF = 0.70), and the proportion of green space inside the circle of influence is the highest (96.7 %) among all the selected stations. Despite its rural nature, the site is reasonably accessible from urban areas within 1 h. Sakakibara and Owa (2005) demonstrated that the more inland rural stations have higher urban heat island intensities than the rural stations near the coast, which affects the evaluation of the urban effect. Thus, they suggest that both urban and rural stations should be located at the same distance from the sea. In this case, the distance from TYW to the sea is almost the same as from HKO (1 km).

According to the results of the LCZ classification, TYW is deemed the most appropriate representative rural station in Hong Kong, based on current weather station network, and TKL can still serve as another rural reference site. However, we also need to take into account the drawbacks of TYW. First, there is a small stream next to the site. Second, the history of TYW is rather short compared to TKL. While TYW has been in operation since 1995, TKL has been in use since 1985 and is one of the earliest automatic weather stations in Hong Kong. Table 6 summarizes and compares the representative urban and rural stations in Hong Kong.

**Table 6:**
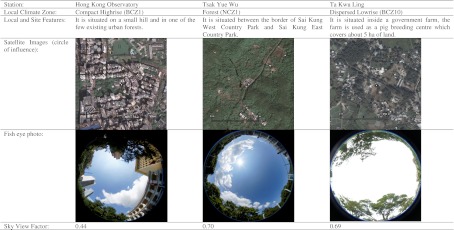
Summary of the representative urban and rural stations in Hong Kong

### Comparison of the temperatures and UHI intensities in Hong Kong

The temperatures and UHI intensities were computed in order to validate our choices of representative weather stations in Hong Kong. The chosen stations include HKO and KP, which were discussed in the section of representative urban station, and CCH, LFS, TKL, HKS, SLW, TYW and WGL, which were discussed in the section of representative rural station.

#### Long-term variations of the temperatures and UHI intensities

**Fig. 3:**
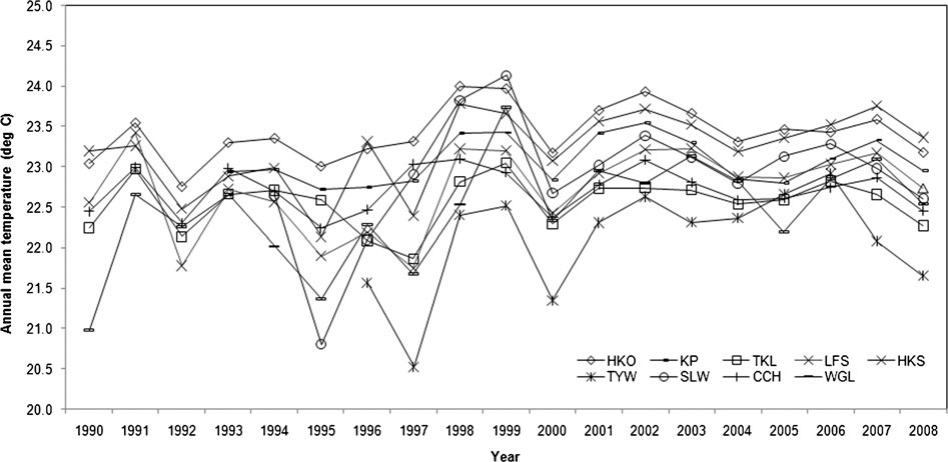
Annual mean temperatures in Hong Kong between 1990 and 2008

**Fig. 4:**
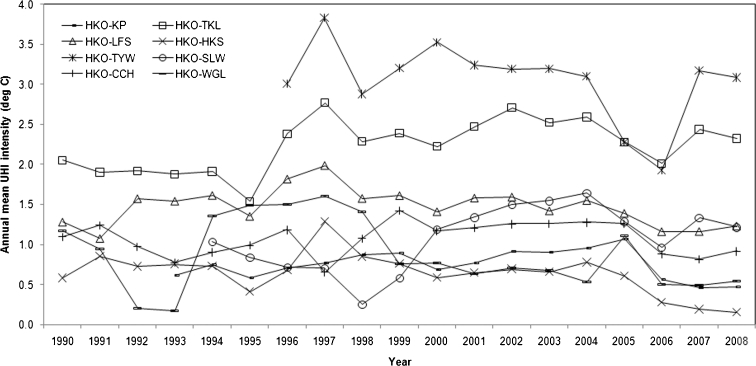
Annual mean UHI intensities in Hong Kong between 1990 and 2008

Twenty years of daily meteorological data were used to calculate the long-term annual mean temperatures and UHI intensities in Hong Kong. Figure 3 shows that the annual mean temperatures at HKO are the highest among all the selected stations in most years and are higher than the temperature at KP every year. Figure 4 illustrates that the annual mean UHI intensity (Δ*T*_HKO-TYW, day_) (i.e., average of all maximum daily UHI intensities for all days within a year) ranges from 1.9 °C in 2006 to 3.8 °C in 1997, and the annual mean UHI intensity (Δ*T*_HKO-TKL, day_) ranges from 1.5 °C in 1995 to 2.8 °C in 1997. None of the other series exceed 2.0 °C across the whole period. This suggests that the HKO station, along with the representative rural sites in Hong Kong, records the representative UHI intensities for the region.

#### Diurnal cycle of the cooling rates and UHI intensities

**Fig. 5:**
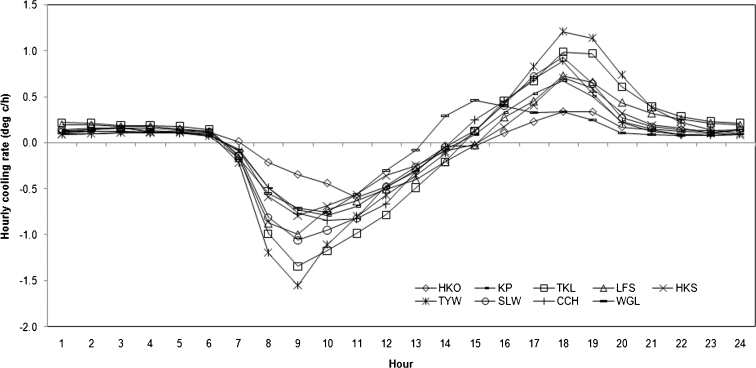
Diurnal variations of the cooling rates in Hong Kong

**Fig. 6:**
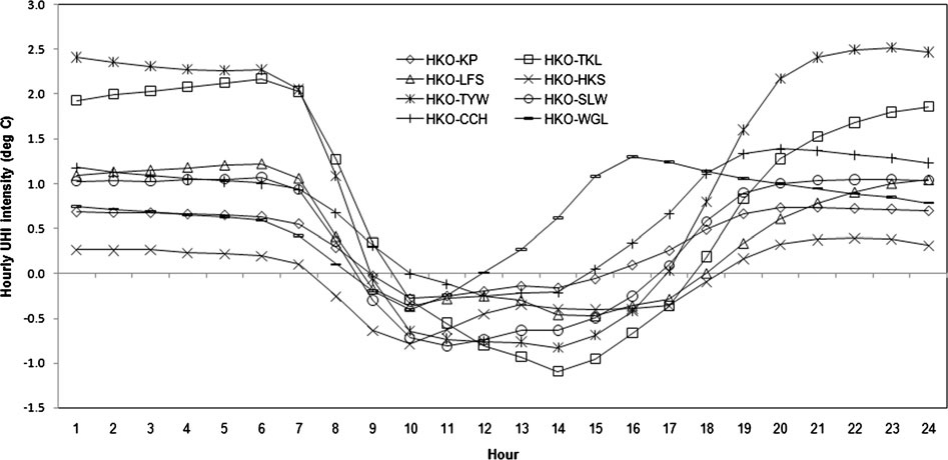
Diurnal variations of the UHI intensities in Hong Kong

Five years of hourly meteorological data were used to determine the diurnal cooling rates and hourly UHI intensities. As Oke (1982) stated, the differences of cooling rates in urban and rural areas drive the diurnal cycle of urban heat island. By averaging all the hourly data, the diurnal variations of the cooling rates and UHI intensities of all seven station pairs are plotted in Figs. 5 and 6, respectively.

In general, TYW and TKL start cooling at 1500 hours, which also marks the beginning of the urban heat island (Fig. 5). Compared to the rural sites, HKO starts cooling 1 h later, which is most probably due to higher thermal conductivities and capacities of urban surfaces. In the urban area, HKO reaches its highest cooling rate (0.3 °C/h) at sunset. The cooling rate then gently declines to 0.1 °C/h and retains that rate until next sunrise. However, simultaneously, the cooling rates at both TYW (1.2 °C/h) and TKL (1.0 °C/h) are higher than those at HKO so that the UHI intensities continue to grow. Figure 6 illustrates that the two station pairs reach their maximum hourly UHI intensities at different times. While HKO-TYW reaches its maximum hourly UHI intensity (Δ*T*_HKO-TYW, h_) (2.5 °C) at 2300 hours, HKO-TKL does not attain its maximum hourly UHI intensity (Δ*T*_HKO-TKL, h_) (2.2 °C) until the sunrise at 0600 hours. The arrival of the maximum UHI intensity at HKO-TKL is delayed due, in large part, to anthropogenic activities surrounding the station that offset the cooling effect during the nocturnal period. After sunrise, as both rural stations start warming rapidly, the urban heat island gradually vanishes at 0900 hours. The urban area usually warms sluggishly because of canyon shading around the urban station (Oke 1982). Afterward, there exists an urban cool island until the rural stations start cooling again.

The other station pairs display somewhat similar diurnal variations; however, their temperature ranges are diminished. Among all the pairs, HKO-WGL is the earliest one to attain its peak UHI intensity, which can be ascribed to the daytime cooling effect from the surrounding sea breeze of Waglan Island.

The values of the warming/cooling rate in Hong Kong are comparable to other tropical cities, but much smaller to the mid-latitude cities. For instance, the mean maximum cooling rates of rural (urban) areas in Hong Kong are 1.2 °C/h (0.4 °C/h), while the values for the tropical city Singapore are 1 °C/h (0.5 °C/h) (Chow and Roth 2006). Under perfect conditions (e.g., no wind and cloud), the nocturnal cooling rates could be 2.8 °C/h (0.8 °C/h) in Montreal, Canada, and 1.8 °C/h (1.0 °C/h) in Seoul, South Korea, respectively (Oke and Maxwell 1975; Lee and Baik 2010).

## Conclusions

In conclusion, the Local Climate Zones system adequately classified all but one of the selected weather stations in Hong Kong, regardless the perplexing surface characteristics of each station. One must be conscious of the challenges during the classification process owing to heterogeneous landscapes. We recommend that the representativeness of stations must be checked case by case, and a field trip to each station should be conducted every year in order to make sure metadata are up to date. One should also avoid classifying a marine station because the current system does not correspond well with this kind of site. In addition, for long-term urban climate studies, we also need to take into account of any plans of urbanization for areas surrounding the current selected stations. For instance, the Hong Kong Government (2009) has implemented a proposal of New Development Area at Ta Kwu Ling with an area of 225 ha, and the development would definitely change the current environment of TKL. In addition, SLW might also be affected by the construction of Hong Kong Section of Hong Kong–Zhuhai–Macao Bridge (Highways Department 2009).

We suggest that HKO and TYW are, respectively, the most appropriate representative urban and rural stations in the current Hong Kong Observatory weather station network. Alternatively, TKL can be employed as another rural reference site. Despite the limited options, Stewart (2011) stated that fewer stations in a representative location are still better than more stations in an atypical location. Finally, we validated our choices by comparing the UHI intensities using different rural sites. The result also quantitatively supports the utilization of the LCZ system. Since the rural stations used in the literature are not representative, the urban heat island intensities in Hong Kong calculated in the previous studies may have been underestimated.
